# Sonic Hedgehog-Induced Histone Deacetylase Activation Is Required for Cerebellar Granule Precursor Hyperplasia in Medulloblastoma

**DOI:** 10.1371/journal.pone.0071455

**Published:** 2013-08-09

**Authors:** Seung Joon Lee, Stephan Lindsey, Bruce Graves, Soonmoon Yoo, James M. Olson, Sigrid A. Langhans

**Affiliations:** 1 Nemours/Alfred I. duPont Hospital for Children, Wilmington, Delaware, United States of America; 2 Fred Hutchinson Cancer Research Center, Seattle, Washington, United States of America; University of Navarra, Spain

## Abstract

Medulloblastoma, the most common pediatric brain tumor, is thought to arise from deregulated proliferation of cerebellar granule precursor (CGP) cells. Sonic hedgehog (Shh) is the primary mitogen that regulates proliferation of CGP cells during the early stages of postnatal cerebellum development. Aberrant activation of Shh signaling during this time has been associated with hyperplasia of CGP cells and eventually may lead to the development of medulloblastoma. The molecular targets of Shh signaling involved in medulloblastoma formation are still not well-understood. Here, we show that Shh regulates sustained activation of histone deacetylases (HDACs) and that this activity is required for continued proliferation of CGP cells. Suppression of HDAC activity not only blocked the Shh-induced CGP proliferation in primary cell cultures, but also ameliorated aberrant CGP proliferation at the external germinal layer (EGL) in a medulloblastoma mouse model. Increased levels of mRNA and protein of several HDAC family members were found in medulloblastoma compared to wild type cerebellum suggesting that HDAC activity is required for the survival/progression of tumor cells. The identification of a role of HDACs in the early steps of medulloblastoma formation suggests there may be a therapeutic potential for HDAC inhibitors in this disease.

## Introduction

Postnatal development of the cerebellum progresses along with the migration and proliferation of neuronal precursor cells which give rise to granule, Purkinje, Golgi, stellate and basket neurons [1]. Among these, the most abundant are cerebellar granular precursor (CGP) cells which originate and migrate tangentially from the rhombic lip of the dorsal hindbrain to form the external germinal layer (EGL). In this layer, CGP cells undergo transient and extensive proliferation. After generating a large pool, they subsequently exit from cell cycle and start to differentiate and migrate along the Bergman glial fibers toward the internal granule layer (IGL) [2]. Granule neurons present the major neuronal population in cerebellum and extend parallel fibers through the Purkinje cell layer into the molecular layer to form synapses with dendrites of Purkinje cells. Purkinje cells are the sole output of the computational circuitry of the cerebellar cortex, but also secrete soluble Sonic hedgehog (Shh) which induces the initial proliferation of CGP cells at the EGL during development [3]. Activation of this pathway is initiated by binding of Shh to the extracellular receptor Patched (Ptch), thereby relieving the inhibition on Smoothened (Smo) allowing for transmission of downstream signals [4]. Mutations of Ptch have been associated with constitutive activation of Smo and are suggested to contribute to an increased onset of medulloblastoma, one of the most common pediatric brain tumors [5]. Underscoring its importance during medulloblastoma progression, mutations in the Shh signaling pathway, including Ptch, Smo and the downstream gene Suppressor of fused (Sufu) have been found in approximately 25–30% of medulloblastoma cases [6].

Several mouse models of medulloblastoma have been developed. These include mice that lack a copy of the Ptch gene [7] or that express a constitutively active mutant of SmoA1 under the control of the NeuroD2 promoter that is active in CGP cells [8]. More sophisticated models limit the expression of these mutations to specific cell types and have significantly contributed to our understanding of the cellular origin of Shh-associated medulloblastoma [9], [10]. While there are still ongoing debates about cellular origin, it seems that both multipotent neural stem cells and the developmentally restricted progenitor cells can give rise to medulloblastoma. Common between those diverse mouse models is an accumulation of CGP cells that fail to migrate inward during development. During normal postnatal development of the cerebellum, CGP cells temporally proliferate in the EGL and then migrate inward to the IGL leaving behind only a small number of foci at the EGL [8], [11]. Medulloblastoma are thought to arise from the surface of the cerebellum where the CGP cells failed to migrate inward properly and continued proliferating. In mice, activation of Shh signals at later stages (after P21) failed to induce medulloblastoma, indicating that inappropriate activation of the Shh pathway per se is not required for tumorigenesis and may just provide the environment for the CGP cells to enter the first stage as a tumor during early development of cerebellum [9]. This suggests a possible therapeutic window for targeted intervention to prevent medulloblastoma development even in the presence of constitutive Shh activation.

Temporal modulation and integration of many genes is essential for coordinating correct timing during development for which chromatin remodeling between open and closed forms plays an important role. In this process, posttranslational modifications of histones such as acetylation, methylation and, phosphorylation, play an important role. In the last decade, histone acetylation has emerged as a crucial regulatory mechanism in various developmental and differentiation processes. Acetylation/deacetylation of histones is regulated by histone acetyltransferases (HATs) and histone deacetylases (HDACs). HDACs catalyze the removal of acetyl groups from lysine residues of histones leading to condensation of chromatin and repression of transcription. Eleven members of classical HDACs have been identified and grouped based on their homology to yeast HDACs [12]. Aberrant expression of HDAC family members has been reported in different types of tumors associated with diverse aspects including metastasis, differentiation and angiogenesis [13]. In medulloblastoma, HDAC5 and HDAC9 up-regulation has been associated with poor prognosis [14] and HDAC inhibitor treatment resulted in down-regulation of the Dickkopf-1 tumor-suppressor through antagonizing Wnt signaling in cultured medulloblastoma cells [15].

In this study, we tested the role of HDACs during the early steps of medulloblastoma transformation, CGP proliferation and CGP accumulation at the EGL during tumor development. Using the Smo/Smo medulloblastoma mouse model, we analyzed the expression patterns of HDACs 1 through 11 and found increased expression and activity of HDACs 1, 2, and 6 in medulloblastoma tumors when compared to wild-type cerebellum or cerebellum from Smo/Smo mice that did not develop tumors. In CGP cells isolated from normal cerebella, Shh-induced proliferation was repressed by trichostatin A, an inhibitor of class I and II HDACs. Consistent with a role of HDACs in CGP proliferation we found that inhibition of HDAC activity *in vivo* during the early postnatal development of the cerebellum blocked the hyperplasia of the EGL in Smo/Smo mice. Although medulloblastoma tumors showed increased HDAC6 expression and activity that was accompanied by reduced tubulin acetylation, the HDAC6-specific inhibitor tubastatin A did not show anti-tumor activity under our experimental conditions. While it remains to be determined whether tubastatin A may enhance drug effectiveness of other chemotherapeutics as has been described for other HDAC inhibitors, our data nevertheless suggest that HDAC inhibitors may be especially beneficial during an early window of therapeutic intervention.

## Materials and Methods

### Lysate Preparation and Immunoblotting

Tissue or cell extracts were prepared in a buffer containing 20 mM Tris-HCl (pH 7.4), 150 mM NaCl, 1 mM EDTA, 0.1% Triton X-100, 1 mM sodium orthovanadate supplemented with protease inhibitor cocktails. Equal amounts of proteins were resolved by SDS-PAGE and transferred to nitrocellulose membrane. Membranes were blocked in 5% skim milk in Tris-buffered saline with 0.1% Tween 20 (TBST), incubated overnight with primary antibody diluted in 5% bovine serum albumin (BSA)/TBST. Antibodies used in these experiments included HDAC1, HDAC2, HDAC3, HDAC4, HDAC5, HDAC6, HDAC7, HDAC9, acetylated α-tubulin, and total α-tubulin (Cell Signaling, Beverly, MA), HDAC10, HDAC11, and acetylated Histone H3 antibodies (EMD Millipore, Billerica, MA) and HDAC8 (Abcam, Cambridge, MA). After incubation with HRP-conjugated secondary antibodies (Cell Signaling) in blocking solution, protein bands were visualized by Enhanced Chemiluminescence Plus (GE Healthcare, Piscataway, NJ).

### HDAC Activity Assay

HDAC activity was measured with the fluorometric HDAC Activity Assay kit (Abcam). Tumor or cell lysates were incubated with assay buffer containing 0.2 mM Boc-Lys(Ac)-AMC as a substrate for 30–60 min. Subsequently, trypsin was added with 1 µM trichostatin A (TSA) to terminate the deacetylation and cleave deacetylated substrates. Fluorescence was measured with the excitation at 390 nm and emission at 460 nm. To measure the activities of individual HDAC members, immunoprecipitation was performed first. Dissected cerebellum or medulloblastoma tissues were lysed in a buffer of 20 mM HEPES (pH 7.4), 150 mM NaCl, 1 mM EDTA, 0.1% Triton X-100, 1 mM sodium orthovanadate, 1 mM PMSF and 5 µg/ml of antipain, leupeptin and pepstatin. Equal amounts of proteins (1–2 mg) were incubated overnight with HDAC-specific antibodies, then 2 hours with protein A-sepharose (GE healthcare, Piscataway, NJ). After extensive washing with immunoprecipitation buffer and twice with HDAC assay buffer (50 mM Tris-HCl, pH 8.0, 137 mM NaCl, 2.7 mM KCl, 1 mM MgCl_2_), HDAC activity assay was performed as described above.

### RNA Extraction and RT-PCR

Total RNA was extracted from wild type cerebellum, healthy cerebellum from Smo/Smo mice, and medulloblastoma tumors obtained from Smo/Smo mice using TRI reagent (Sigma, St Louis, MO) according to the manufacturer’s instructions. First-strand cDNA was synthesized from 1 µg of RNA using the iScript cDNA Synthesis kit (Bio-Rad, Hercules, CA). Quantitative PCR analysis was performed with a SYBR Green PCR master mix (Applied Biosystems, Foster City, CA) using an ABI Prism 7900 Sequence Detection System (Applied Biosystems, Foster City, CA) and normalized to β2-microglobulin. All primer sequences used for qPCR analyses are included in Table S1.

### Primary Culture of Cerebellar Granule Precursor (CGP) Cells

Cerebellum was dissected from P4-6 pups of either wild type or Smo/Smo mice and dissociated into single cells using the Papain Dissociation System kit (Worthington Biochemical Corp, Freehold, NJ). After filtering through a nylon mesh (70 µm pore size), the cell suspension was preplated twice to remove astroglial cells and fibroblasts. CGPs were cultured in poly D-lysine coated dishes in Neurobasal medium supplemented with 0.25 mM KCl and B27. Medium was replaced every other day. Shh (3 µg/ml) or SAG (0.1 µM, 1 µM) was added 30 min after plating.

### Cell Proliferation Assay

Proliferation of CGP cells treated with SAG and HDAC inhibitors (TSA, Tubastatin A) was measured by CellTiter-Blue Cell Viability Assay (Promega, Madison, WI). Three and six days after incubation in 96 wells, 20 µl of CellTiter Blue reagent was added and incubated for 4 hours. Conversion of a redox dye (reaszurin) into a fluorescent resorufin by living cells was quantified using a Victor X4 multilabel plate reader (Perkin-Elmer, Waltham, MA). Values represent the mean ± S.E. from four determinations.

### Histology and HDAC Inhibitor Treatment of Smo/Smo Mice

TSA (Sigma, St Louis, MO) and tubastatin A (Cayman Chemical, Ann Arbor, MI) were dissolved in DMSO to yield 20 mg/ml. 7-day old Smo/Smo mice were subcutaneously injected with DMSO, TSA (5 mg/kg body weight) or tubastatin A (5 mg/kg body weight) diluted in phosphate-buffered saline daily for two weeks. Brains were dissected, fixed with formalin, and embedded in paraffin. One of every 10 sections from 10 µm serial sections was deparaffinized, rehydrated, and stained with hematoxylin and eosin. The thickness of the EGL was measured after acquiring images under the 20× objective of a Nikon Eclipse 80i microscope. Additional tissue sections were incubated with antibodies toward acetylated α-tubulin (Cell Signaling) and visualized. This study was carried out in accordance with the recommendations in the Guide for the Care and Use of Laboratory Animals of the National Institutes of Health and was approved by the Nemours Institutional Animal Care and Use Committee.

## Results

### HDAC Expression in Medulloblastoma

**Figure 1:**
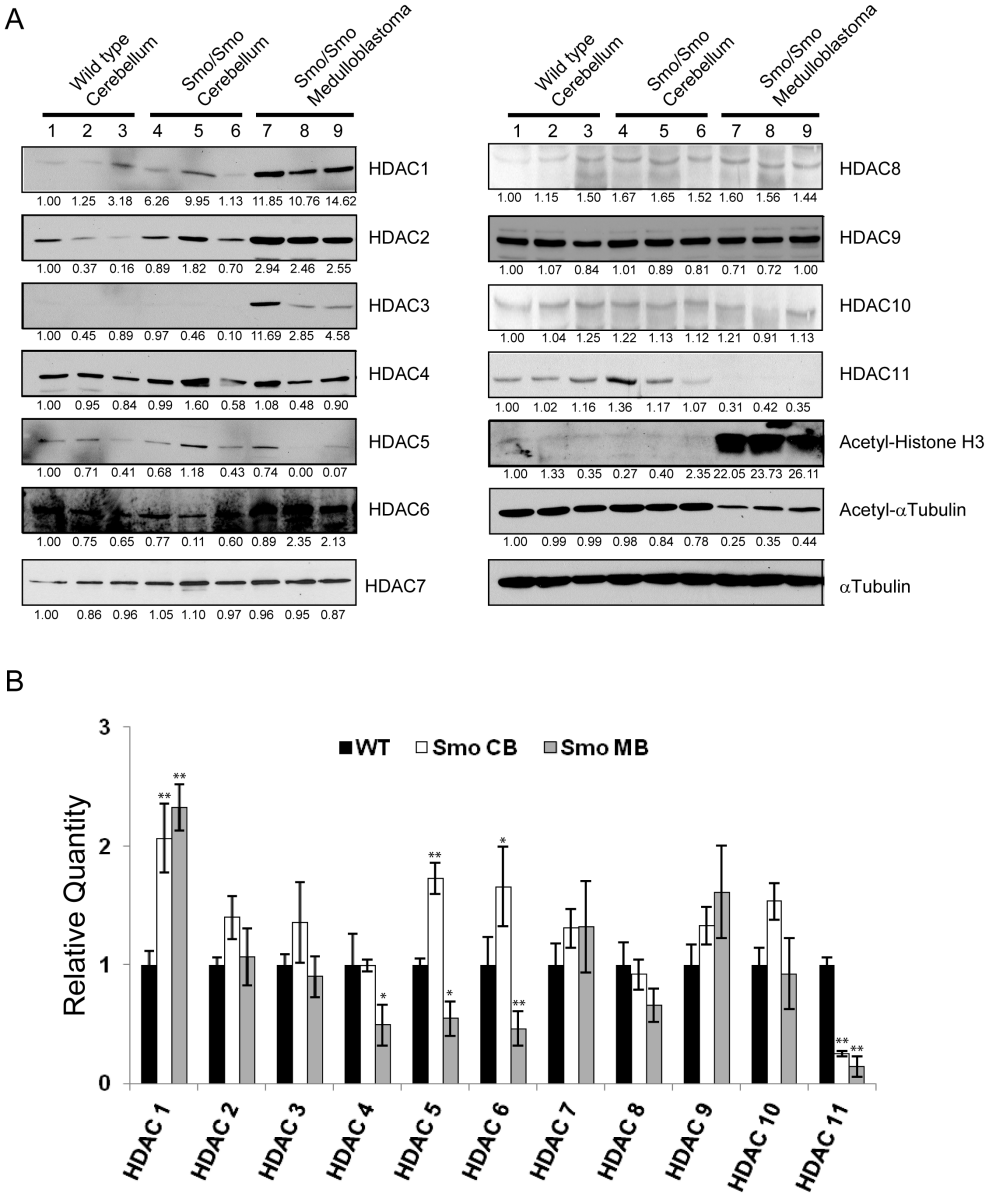
HDAC protein and mRNA levels in medulloblastoma. A, Immunoblot of HDACs 1 through 11 in lysates from wild type cerebellum, Smo/Smo cerebellum and medulloblastoma. Numbers below immunoblots are the intensity normalized to αTubulin. B, Relative quantity of individual HDAC members were measured by qRT-PCR from wild type cerebellum (WT), Smo/Smo cerebellum (Smo CB), and Smo/Smo medulloblastoma (Smo MB) (n = 7). Bars correspond to mean ± S.E. *P<0.05. **P<0.0001.

Several individual HDAC family members have been suggested to play a role in cancers of various origins. In medulloblastoma patients, aberrant expression of HDAC5 and HDAC9 have been correlated with poor clinical outcome [14]. In an effort to further understand the role of HDACs in medulloblastoma development, we compared protein and mRNA levels of individual HDAC family members in lysates obtained from wild type cerebellum to healthy cerebellum and medulloblastoma tumors from Smo/Smo transgenic mice. Smo/Smo mice express a constitutively active Smo mutant protein resulting in extended proliferation of CGPs. Eventually up to 94% of mice develop medulloblastoma within two months [16] but the time when individual mice show anatomical and behavioral symptoms of tumors may vary from two months to a year. Thus, in addition to tumors from Smo/Smo mice that had developed medulloblastoma, we isolated cerebellum from mice that did not exhibit symptoms or display abnormalities in gross morphology ranging in age from four months to one year. Using immunoblotting and real time-PCR we compared protein (Fig. 1A) and mRNA (Fig. 1B) profiles, respectively, of various HDAC family members. We found an increase in HDAC1 protein and mRNA levels in Smo/Smo cerebellum and Smo/Smo medulloblastoma as compared to wild-type cerebellum. HDAC2 and HDAC3 protein, but not mRNA levels, were increased in Smo/Smo tumors but not in cerebellum from normal mice or Smo/Smo mice without tumors. While we did not find any apparent changes in HDAC4 protein levels, HDAC4 mRNA was reduced in tumors but not Smo/Smo cerebellum. HDAC5 protein and mRNA levels were lower in tumors when compared to cerebellum and HDAC11 protein and mRNA levels were reduced in tumors. HDAC6, albeit decreased mRNA level, showed increased expression in tumors. HDAC7, HDAC8, HDAC9, and HDAC10 did not reveal any significant differences in protein or mRNA levels between wild-type cerebellum, healthy Smo/Smo cerebellum, or Smo/Smo tumors. Consistent with increased HDAC6 expression and tubulin being one of the primary substrates of HDAC6, tubulin acetylation was decreased in tumors when compared to normal and Smo/Smo cerebellum. Together, these results suggest not only a complex regulation of various HDAC family members during medulloblastoma development but also that possibly not only HDAC1 but other HDACs, such as HDAC2, 3, or 6 might contribute to tumor development.

### Increased HDAC Activity in Medulloblastoma

**Figure 2:**
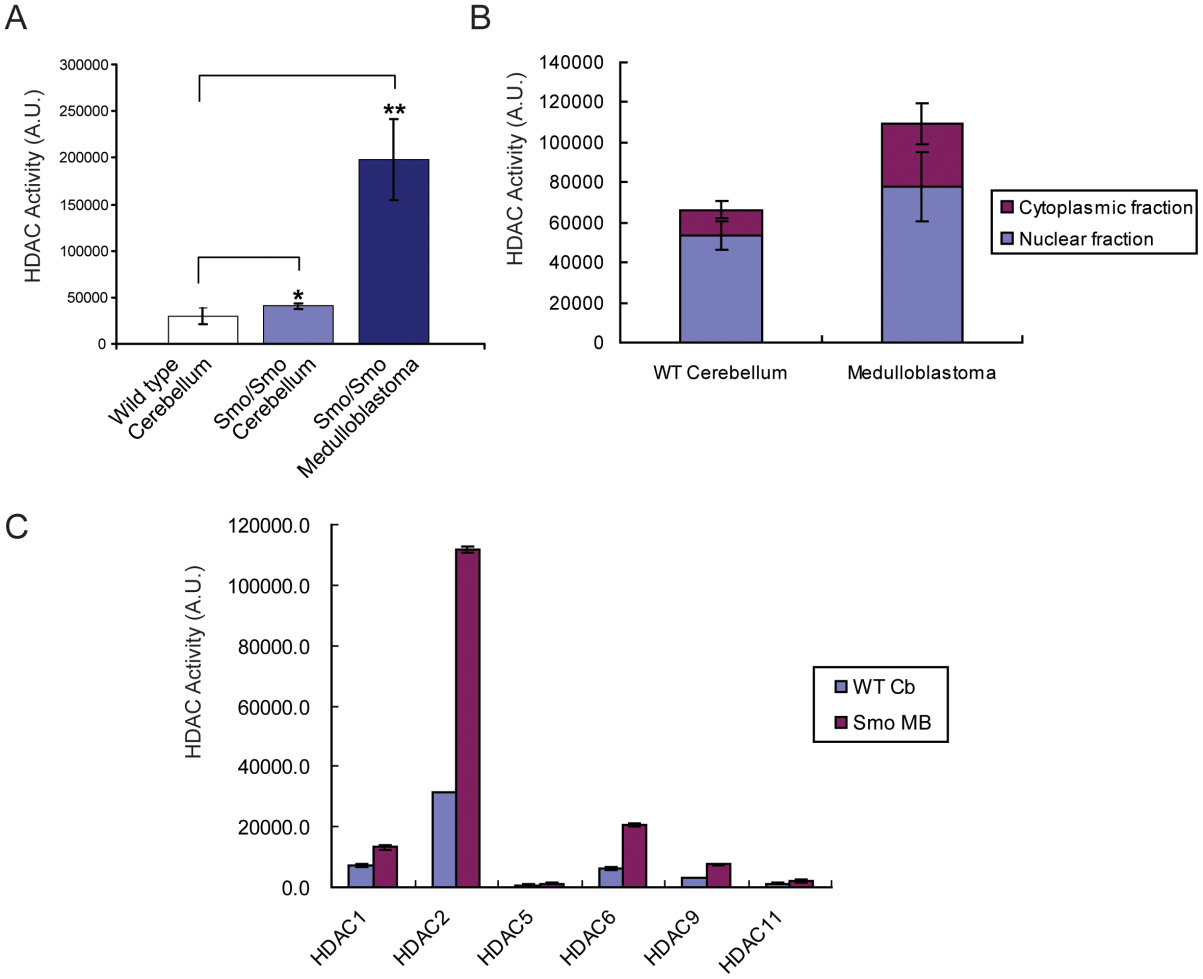
Increased HDAC activity in medulloblastoma. A, HDAC activity was measured from wild type and Smo/Smo mice cerebellum and compared with medulloblastoma from Smo/Smo mice. *P = 0.073. **P<0.005. B, HDAC activity was measured from cytoplasmic and nuclear fractions of wild type cerebellum and Smo/Smo medulloblastoma. C, HDAC activity of individual HDAC members were measured after immunoprecipitation with specific antibodies. Bars correspond to mean ± S.E. Other HDAC members (HDAC3, 4, 7) did not exhibit any significant activity above the background.

To test whether the changes in expression patterns of individual HDAC members altered the overall HDAC activity in tumors, we first measured the total HDAC activity in cell lysates from Smo/Smo mouse tumors and cerebella of normal mice and Smo/Smo mice that did not display any symptoms. Using a fluorimetric HDAC activity assay we found that the total HDAC activity was about five times higher in medulloblastoma compared to normal cerebellum (Fig. 2A). A slightly higher activity was measured in Smo/Smo mouse cerebellum compared to wild type cerebellum, albeit not statistically significant (P = 0.073). Immunoblotting with anti-acetylated lysine antibody did not show a striking difference in the overall acetylation pattern of medulloblastoma and normal cerebellum (Fig. S1) except for the absence of a prominent band of about 50 kDa in tumor lysates that was confirmed to be acetylated tubulin (see below). It is reasonable to assume that the increased HDAC activity in medulloblastoma may be associated with the regulation of a more specific subset of genes associated with tumor proliferation. Immunoblotting for various known cell cycle regulators revealed an increase in cyclin B1, cyclin D1, p27 Kip1 and p21 Waf1/CIP1 and in the mitotic marker phospho-histone H3 (Fig. S1). Thus, activation of HDACs in medulloblastoma may play a role in maintaining the high proliferative capacity of these tumors.

Depending on the isoform, HDACs can be localized to either the cytoplasm or the nucleus or may shuttle between the two compartments. Measurement of the HDAC activity in cytosolic and nuclear fractions of wild type cerebellum and tumors of Smo/Smo mice revealed increased HDAC activity in both the cytoplasm and nuclei of Smo/Smo medulloblastoma tumors (Fig. 2B). This suggested that not only the nuclear HDAC1 and HDAC2 that had shown the highest increase in protein levels in tumors (Fig. 1A) but also other HDAC family members that can be found in the cytosol (e. g. HDAC6) might have contributed to the observed increase in total HDAC activity in medulloblastoma. Therefore, immunoprecipitation using specific antibodies determined the activity of individual HDAC family members and found that HDAC1, 2, 6 and 9 had elevated activity in tumor lysates compared to normal cerebellum (Fig. 2C). HDAC5 and HDAC11 did not reveal any difference and the activities of other members were not in the detectable range. Comparison of the half maximal effective concentration (EC50) against the HDAC inhibitor, Trichostatin A (TSA) that exhibits more potency toward class I family HDACs (HDAC1, 2, 3) than class IIa (HDAC4, 5) [17], [18] revealed an EC50 of 16.44 nM in tumors and of 31.11 nM in wild-type cerebellum (Fig. S2) suggesting that HDAC1 and 2 might be the predominant contributors to the increased HDAC activity observed in Smo/Smo tumors.

### Altered HDAC Expression and Activity during CGP Cell Differentiation

**Figure 3:**
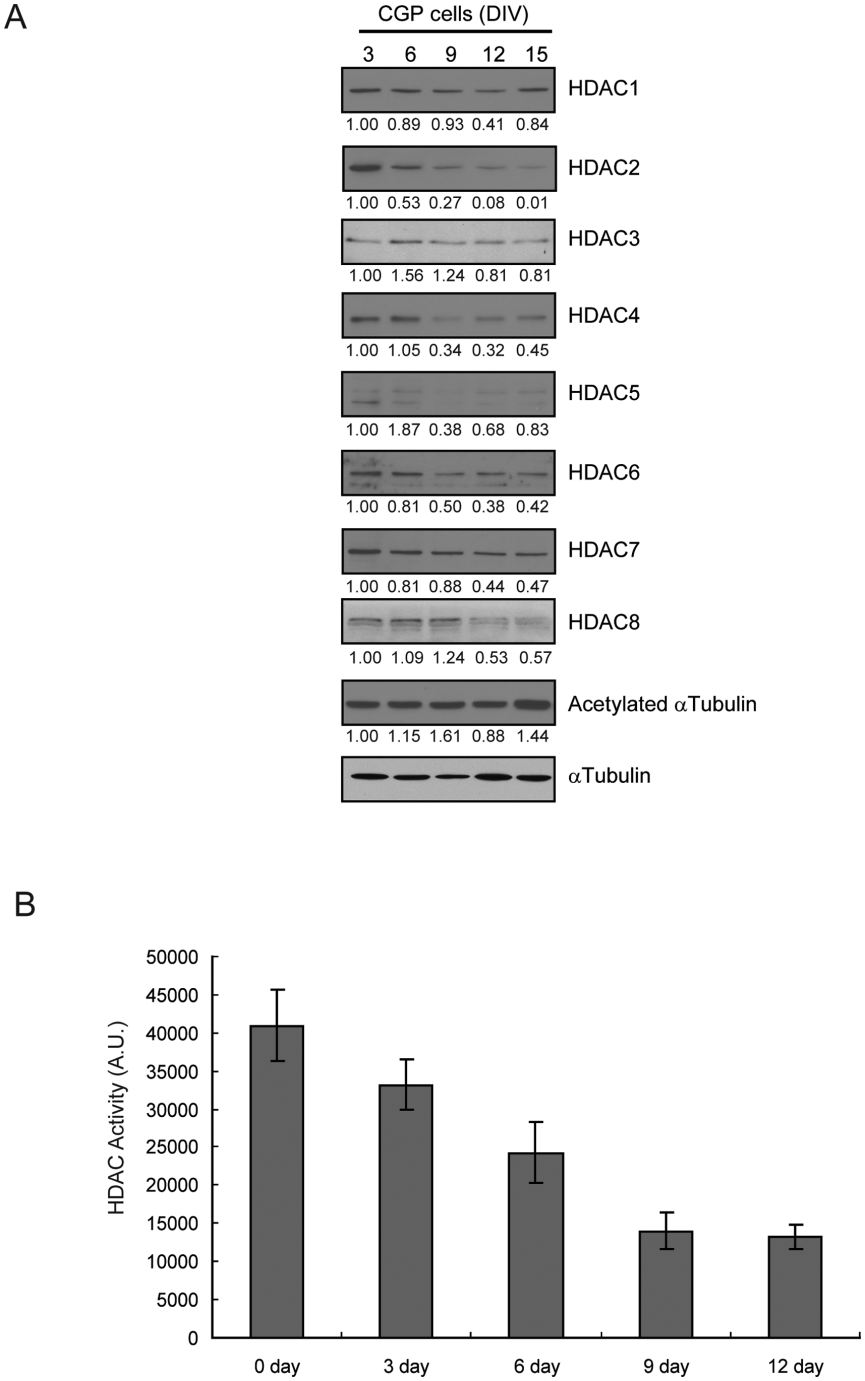
Differentiating CGP cells show a decline of HDAC activity and protein level. A, Immunoblot of CGP cells differentiating in vitro. CGP cells isolated from P5 wild type mice were plated and harvested at different days of in vitro culture (DIV). Numbers below immunoblots are the intensity normalized to undifferentiated cells. B, HDAC activity of CGP cells differentiated in vitro was measured. Assay was performed three times in triplicate and the representative one is shown. Bars correspond to mean ± S.E. of three independent experiments.

The postnatal development of cerebellum can be divided into an active proliferation stage and a subsequent differentiation/synaptogenesis period. Deregulated proliferation of CGP cells is thought to contribute to tumor formation in medulloblastoma. Shh released from Purkinje neurons plays an important role in controlling proliferation and differentiation of CGP cells and thus, we investigated the relationship between Shh-induced CGP proliferation/differentiation and HDAC activity. First, we isolated CGP cells from wild type mice that spontaneously differentiate into granule neurons when cultured *in vitro* and analyzed the HDAC expression pattern at various time points. As the CGP cells differentiated we found a gradual decrease in expression of various HDAC isoforms, including HDAC2, 4, 5, 6, and 7 and an increase in tubulin acetylation (Fig. 3A). Decreased protein expression of these HDACs was accompanied by reduced total HDAC activity (Fig. 3B) suggesting that HDAC function may be regulated during CGP differentiation.

**Figure 4:**
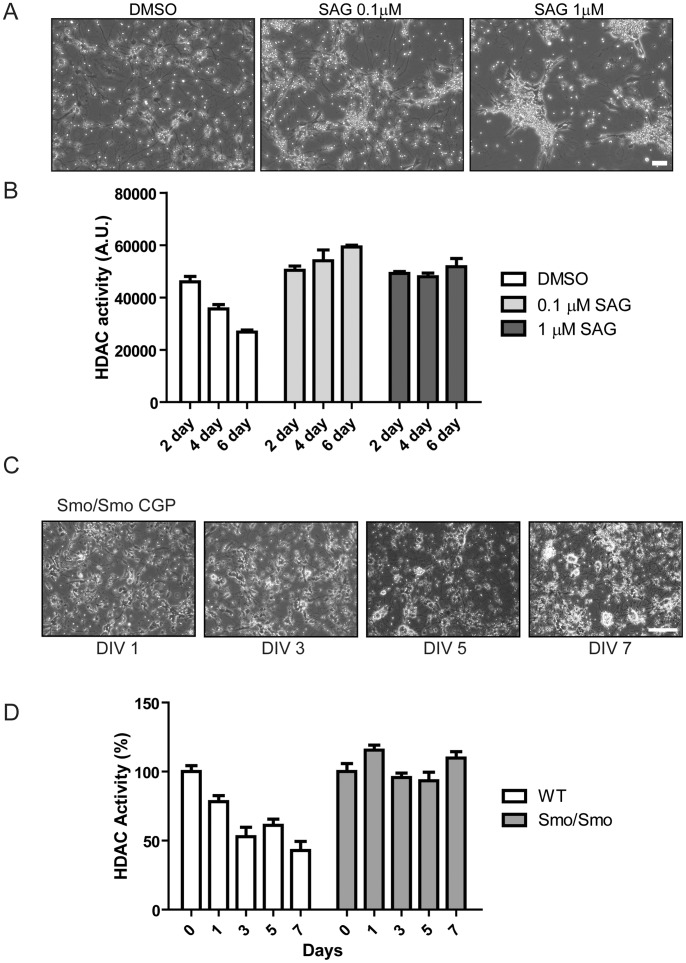
Shh induced HDAC activation and proliferation in CGP cells. A, Phase contrast image of CGP cells incubated with DMSO, 0.1 µM SAG or 1 µM SAG for 6 days. Scale bar represents 100 µm. B, HDAC activity was measured from CGP cells incubated either with DMSO or different concentrations of SAG for 2, 4 and 6 days. Bars correspond to mean ± S.E. C, Phase contrast images of CGP cells from Smo/Smo mice after 1,3,5 and 7 days in vitro culture. Scale bar represents 100 µm. D, HDAC activity was measured from cultured CGP cells isolated from wild type and Smo/Smo mice. Bars correspond to mean ± S.E.

Treatment with Shh or a synthetic Shh agonist (SAG) strongly induced proliferation of CGP cells isolated from wild type mice (Fig. 4A) [3], and induced expression of Gli-1, a hallmark of activation of the Shh signaling cascade (Fig. S3). As control, in CGP cells differentiated in culture, total HDAC activity gradually decreased over time (Fig. 4B). In contrast, SAG-treatment of CGP cells resulted in increased HDAC activity that remained elevated compared to mock-treated CGP cells even after 6 days in culture (Fig. 4B). When CGP cells were isolated from Smo/Smo mice they proliferated *in vitro* even in the absence of Shh or Shh agonist (Fig. 4C). As opposed to differentiating CGP cells isolated from wild-type mice in which HDAC activity decreased over time, the total HDAC activity remained high in CGP cells isolated from Smo/Smo mice (Fig. 4D). Taken together, these results show that Shh-induced proliferation of CGP cells is accompanied by sustained activation of HDAC, suggesting that HDAC function may be involved in controlling the proliferation and differentiation of CGP cells.

**Figure 5:**
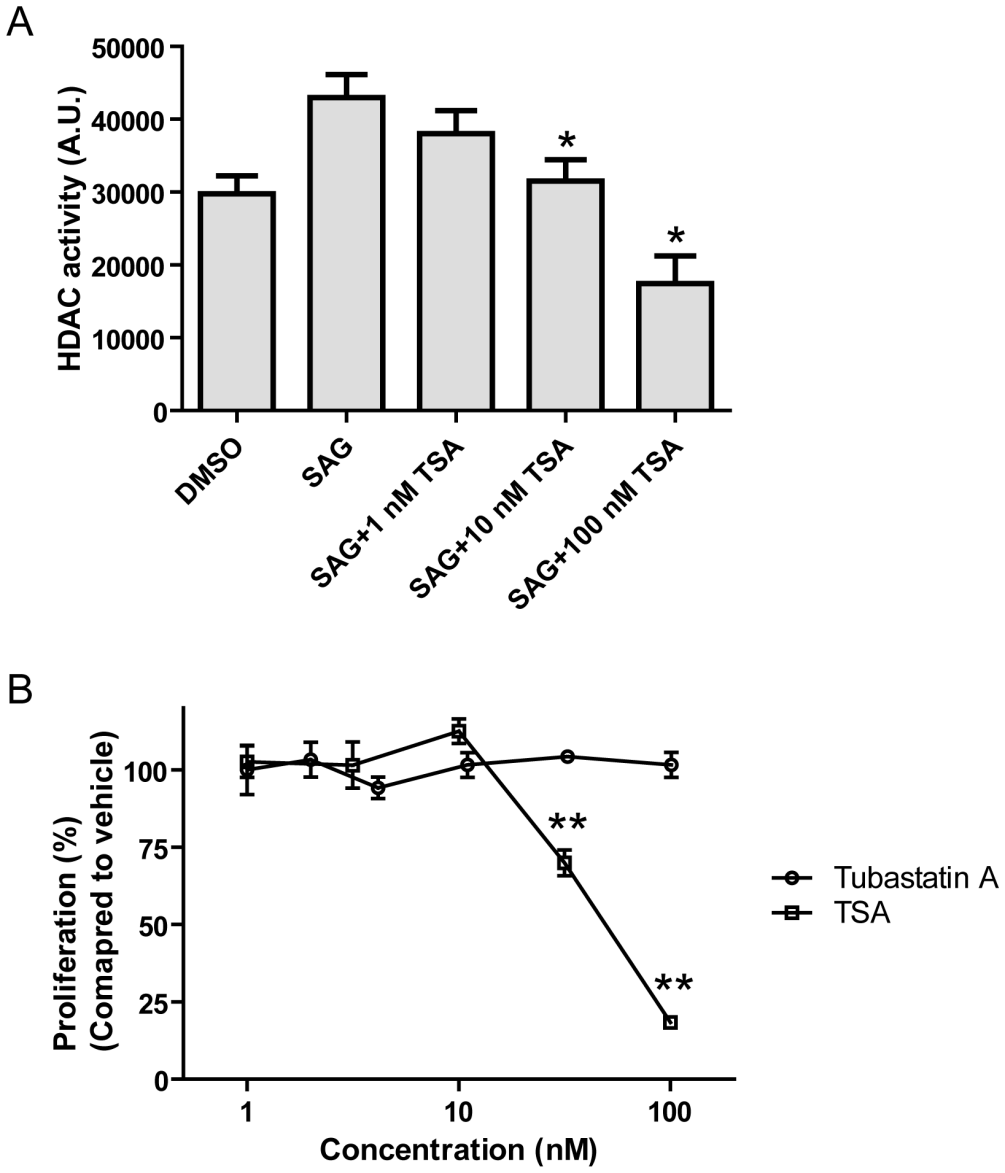
Inhibition of HDAC blocks Shh-induced proliferation. A, HDAC activity was measured from CGP cells treated with increasing concentrations of TSA along with 0.1 µM SAG for 3 days. Bars correspond to mean ± S.E. *P<0.05. B, Proliferation of CGP cells induced by 0.1 µM SAG were measured with increasing concentrations of Tubastatin A or TSA. Data were expressed relative to the proliferation rate of DMSO-treated control cells. Error bars represent mean ± S.E. **P<0.001.

To determine whether high HDAC activity is required for granule cell proliferation, we tested whether Shh (SAG)-induced proliferation of CGP cells can be blocked by HDAC inhibitors. Since various HDAC inhibitors have been reported to induce cell death in medulloblastoma cell lines [19], we used TSA, a pan-HDAC inhibitor at relatively low concentrations (10 nM∼100 nM) that reduced SAG-induced HDAC activation close to levels of unstimulated (differentiating) CGP cells but did not induce cell death (Fig. 5A). In addition, since we observed increased HDAC6 protein levels (Fig. 1A) and activity (Fig. 2C) in Smo/Smo tumors, we also included the HDAC6-specific inhibitor Tubastatin A [20] in our studies. Treatment with TSA but not Tubastatin A blocked SAG-induced CGP proliferation (Fig. 5B). Thus, our data suggest that HDAC activity is required to promote CGP proliferation and is most likely mediated by class I HDACs and not HDAC6.

### HDAC Inhibition Blocks Accumulation of Preneoplastic Cells at the EGL

**Figure 6:**
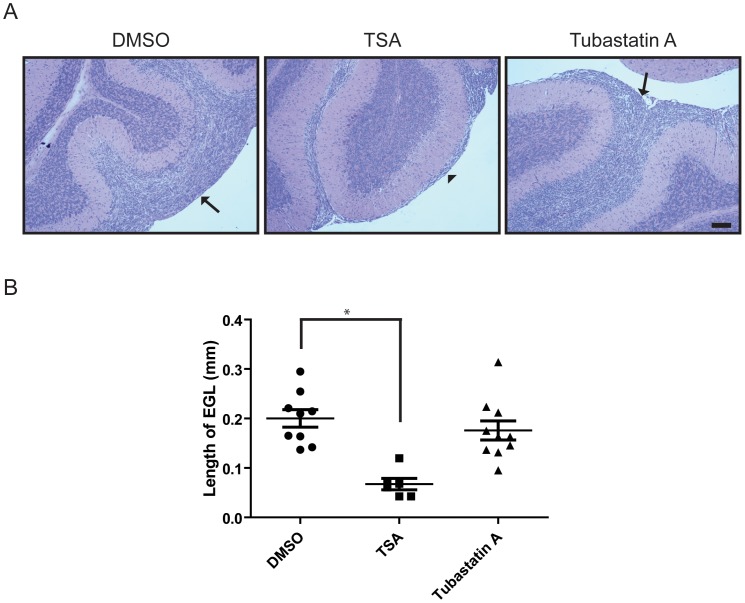
Inhibition of HDAC ameliorates hyperplasia of CGP cells during postnatal cerebellum development. A, Bright field images of paraffin-embedded mice brain sections that were stained with hematoxylin and eosin. Smo/Smo mice were injected with DMSO, TSA or Tubastatin A for two weeks and then dissected brains were fixed and processed for staining. Arrows indicate hyperplasia of CGP cells after DMSO (control) or Tubastatin A treatment and arrowhead indicates the narrow EGL after TSA treatment. Scale bar represents 100 µm. B, Plot showing the length of EGL from DMSO, TSA or Tubastatin A injected mice. Each symbol represents average length of individual mice and mean ± S.E. were shown. *P<0.0001.

During early postnatal development of the cerebellum, CGP cells undergo transient and extensive proliferation in the EGL, subsequently exiting from the cell cycle and migrating towards the IGL. Constitutive activation of Shh signaling is thought to induce abnormal CGP proliferation and accumulation of preneoplastic cells in the EGL, which then presumably develop into medulloblastoma tumors. We thus tested whether pharmacological inhibition of HDAC activity with TSA or Tubastatin A could suppress the accumulation of preneoplastic cells in the EGL of Smo/Smo mice. Both TSA and Tubastatin A have been shown to pass the blood-brain barrier and show efficacy in different mouse models [21], [22], [23]. At the age of around three weeks CGP cells form distinctive layers in the cerebellum in wild-type mice (data not shown), but in Smo/Smo mice CGP cells remained in the EGL forming a thick layer of cells at the outer cortex (Fig. 6A). The average thickness of the EGL in vehicle-treated Smo/Smo mice was found to be around 200 µm (Fig. 6A, B). In TSA-treated animals the average thickness of the EGL was significantly reduced to around 60 µm (Fig. 6A, middle panel, arrowhead) and the IGL was well-developed indicating that inward migration had occurred. Consistent with our *in vitro* data in primary cultures of CGP cells, Tubastatin A-treatment had no significant effect and the thickness of the EGL was comparable to vehicle-treated mice (Fig. 6A, B), although tubulin acetylation as a measure for HDAC6 inhibition was increased (Fig. S4). It remains to be determined whether in combination with other drugs tubastatin A might enhance drug effectiveness as has been described for other HDAC inhibitors. Together, our data suggest that HDAC activity is required for sustained proliferation of CGP cells *in vivo*. Furthermore, we show that that pharmacological inhibition of HDAC activity during early postnatal development of the cerebellum could block Shh-induced CGP hyperplasia in the Smo/Smo mice suggesting that HDAC inhibitors may be especially beneficial early on or in very young children for therapeutic intervention in medulloblastoma.

## Discussion

HDACs exhibit great promise as potential drug targets in medulloblastoma and various HDAC inhibitors have shown anti-tumor effects in pre-clinical tumor models [19]. However, the roles of HDACs in early stages of tumor cell commitment and medulloblastoma initiation are not well-characterized. By current consensus, medulloblastoma can be divided into four molecular subsets, one of which is characterized by aberrant activation of the Shh signaling cascade [24]. Here we show that Shh signaling activates HDACs to induce proliferation of CGP cells, which are thought to be the cells of origin in this subset of tumors. In a preclinical model of Shh-induced medulloblastoma, HDAC expression and activity of various HDAC family members, including HDAC1, 2, 3 and 6 were increased in tumors. While pharmacological inhibition of HDAC activity with the pan-inhibitor TSA abolished Shh-induced CGP proliferation *in vitro* and *in vivo*, the HDAC6-specific inhibitor Tubastatin A was not effective in inhibiting cell proliferation. However, it remains to be determined whether increased HDAC6 activity contributes to medulloblastoma pathogenesis in ways other than proliferation, eg tumor cell commitment or tumor metastasis. Together our studies suggest that HDAC activation is required for initiation and maintenance of Shh-induced medulloblastoma and warrant the development of HDAC inhibitors for the treatment of these tumors. We further suggest that in the light of some medulloblastoma developing rapid resistance to Shh inhibitors, a combination of HDAC inhibitors and Shh inhibitors might prove advantageous to effectively target these tumors.

While it is not known whether specific HDAC isoform profiles are associated with distinct medulloblastoma subtypes, various studies in human medulloblastoma tissues and in medulloblastoma mouse models reported changes in HDAC expression. In constitutively activated SmoM2 mice (GDS3008,3009) HDAC2 and HDAC9 were increased. HDAC2 levels were also elevated in medulloblastoma when compared to CGP or preneoplastic regions of patched mice [11]. Another study reported that HDAC1 mRNA and protein levels were upregulated in mouse Ptch1^+/−^ medulloblastoma [25]. In human medulloblastoma, HDAC5 and HDAC9 upregulation were identified as markers for tumors with poor prognosis [14]. In our current study of Smo/Smo tumors we found increased HDAC1 and HDAC 2 protein levels (Fig. 1A) that were accompanied by increased HDAC1 and HDAC2 activity (Fig. 2C) but neither HDAC5 nor HDAC9 were upregulated. While HDAC2, HDAC3 and HDAC6 showed increased protein levels, the RNA levels either did not change (HDAC2 and HDAC3) or even decreased (HDAC6) in tumor samples. At this point we do not know whether an auto-regulatory loop as described for HDAC1 [25] exists for other HDACs as well, and thus may explain the discrepancy between the mRNA and protein levels of individual HDAC family members. We consistently observed increased HDAC6 protein expression and activity in Smo/Smo tumors (Figs. 1A and 2C). High HDAC6 protein expression levels were accompanied by decreased tubulin acetylation (Fig. 1A), one of the major substrates of HDAC6. In addition, HDAC6 protein expression decreased and tubulin acetylation increased as CGP cultures differentiated (Fig. 3). This suggested that HDAC6 and tubulin acetylation are developmentally regulated and that HDAC6 dysregulation could be associated with medulloblastoma pathogenesis. Nevertheless, the HDAC6 specific inhibitor Tubastatin A did not affect CGP proliferation *in vitro* (Fig. 5B) or *in vivo* (Fig. 6). At this point, it is not known whether abnormal HDAC6 expression and activity contributes to medulloblastoma progression overall, or possibly during a specific time window in early or post-natal cerebellum development. Interestingly, HDAC11 protein and mRNA levels were reduced drastically in Smo/Smo medulloblastoma. HDAC11 is thought to associate with HDAC6 and studies on the significance of this finding are in progress. In contrast, overall inhibition of HDAC activity with the non-specific inhibitor TSA reduced CGP proliferation both in culture and in Smo/Smo mice (Figs. 5 and 6). We further observed consistently increased HDAC1 and HDAC2 expression and activity in Smo/Smo tumors and decrease in differentiating CGP cells. While further studies will be required to delineate the roles of individual HDACs in tumor pathogenesis, we suggest that HDACs are valid targets for drug development for medulloblastoma.

Initiation and progression of medulloblastoma is intimately linked to signaling cascades including Shh, Wnt, and Notch that play important roles in postnatal cerebellar development. During this period, these pathways regulate the proliferation and differentiation of stem and progenitor cells that originate from the rhombic lip or ventricular zone [26]. Aberrant activation of these cascades is closely associated with medulloblastoma and alterations in each pathway can give rise to distinct subtypes of tumor [27]. Among them, mutations and deletions of the Shh pathway components PTCH1, Suppressor of fused (SUFU), and RENKDCT11 [24] have been implicated in medulloblastoma. One of the major downstream effectors of Shh signaling is the transcriptional activator Gli (glioma-associated oncogene) and the activity of Gli1 and Gli2 depends on HDAC1-mediated deacetylation by Shh [25]. We found that in Smo/Smo mice with constitutive activation of Shh signaling not only CGP cells continued to proliferate in the EGL, but HDAC1 and HDAC2 expression and activity were high. Similarly, in primary CGP cultures isolated from normal cerebellum, pharmacological activation of Shh signaling not only sustained cell proliferation but also HDAC activity while in untreated cells HDAC activity decreased with subsiding proliferation and increasing differentiation, an effect that was not observed in CGP cells isolated from Smo/Smo mice. In agreement with our hypothesis that HDAC activity is critical for Shh-mediated proliferation, in our studies inhibition of HDAC activity abolished Shh-induced proliferation both *in vitro* and *in vivo.* Similarly, the HDAC inhibitor SAHA exhibited anti-tumor activity toward both a xenograft model and transgenice Smo/Smo mice alone or in combination with retinoic acid [28]. HDACs have been suggested to be involved in cancer initiation/progression through regulation of the expression and activity of numerous proteins associated with cell cycle regulation, proliferation, differentiation, and apoptosis. Inhibitors against HDACs are thought to induce transcription of dormant cell cycle regulators through increasing the accessibility of chromatin [29]. However, our studies suggest that HDACs may not only regulate cell proliferation at the chromatin level but contribute to tumor formation by playing an integral role in oncogenic signaling cascades such as the Shh signaling pathway. It remains to be determined whether HDACs also could contribute to medulloblastoma development of a different subclass of tumors.

## Supporting Information

Figure S1 Immunoblot of acetylated proteins and cell cycle regulators from lysates of wild type cerebellum, Smo/Smo cerebellum and Smo/Smo medulloblastoma.
(TIF)

Figure S2 HDAC activity was measured from wild type cerebellum, Smo/Smo cerebellum and Smo/Smo medulloblastoma with increasing concentration of TSA. Error bars represent S.E.
(TIF)

Figure S3 Immunoblot for Gli1 in CGP cells treated with either DMSO or SAG for indicated concentration and time. A GAPDH immunoblot was included as loading control.
(TIF)

Figure S4 Immunhistochemistry for acetylated tubulin in brain sections from Smo/Smo mice injected with DMSO, TSA or Tubastatin A.
(TIF)

Table S1 (DOCX)
