# Supplementary figures and images for: Sonic Hedgehog-Induced Histone Deacetylase Activation Is Required for Cerebellar Granule Precursor Hyperplasia in Medulloblastoma
Source: PLoS One. 2013 Aug 9;8(8):e71455. doi: 10.1371/journal.pone.0071455 (PMC3739791; doi:10.1371/journal.pone.0071455)

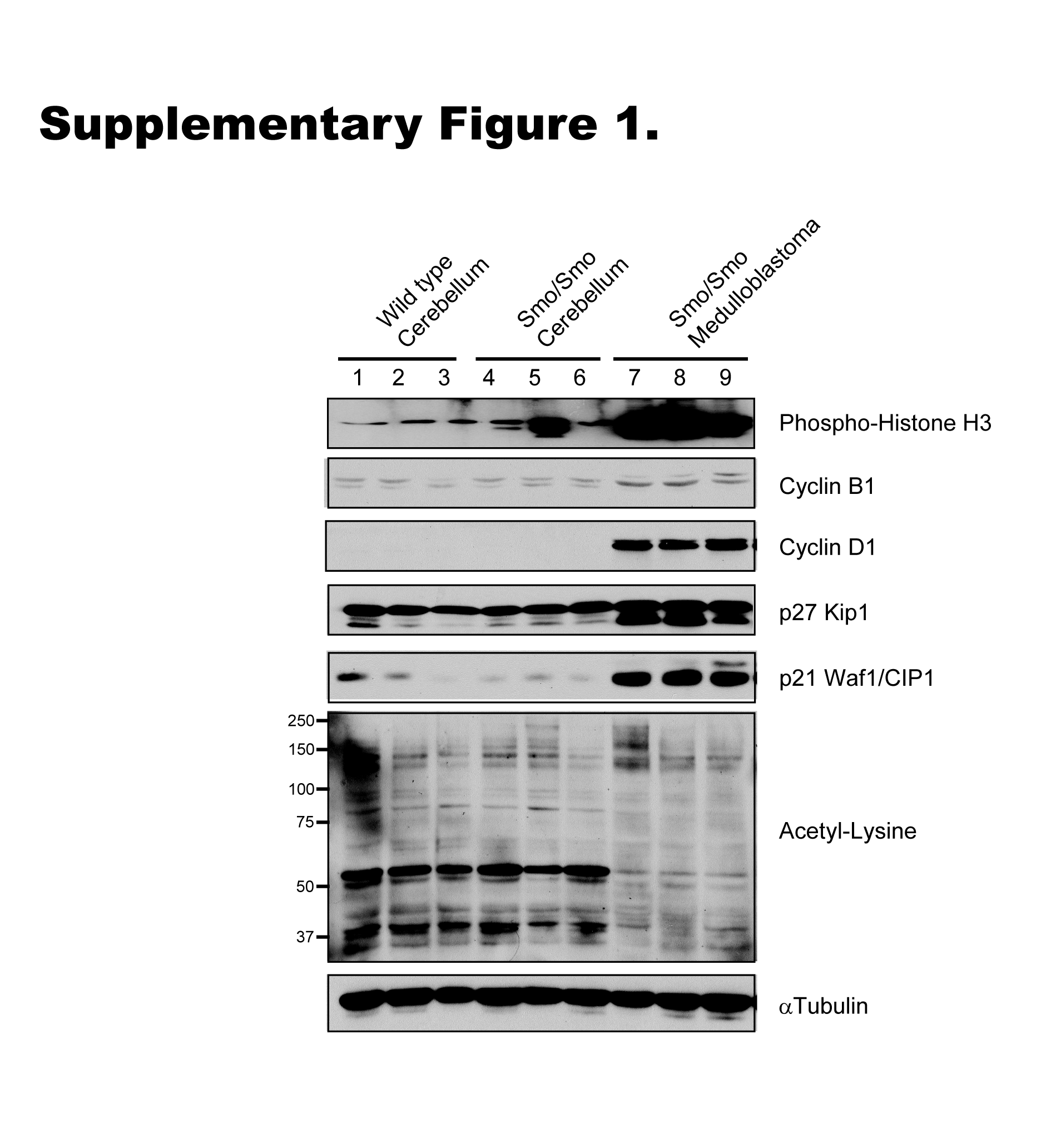

Supplement: Figure S1 — Immunoblot of acetylated proteins and cell cycle regulators from lysates of wild type cerebellum, Smo/Smo cerebellum and Smo/Smo medulloblastoma. (TIF) [file pone.0071455.s001.tif]

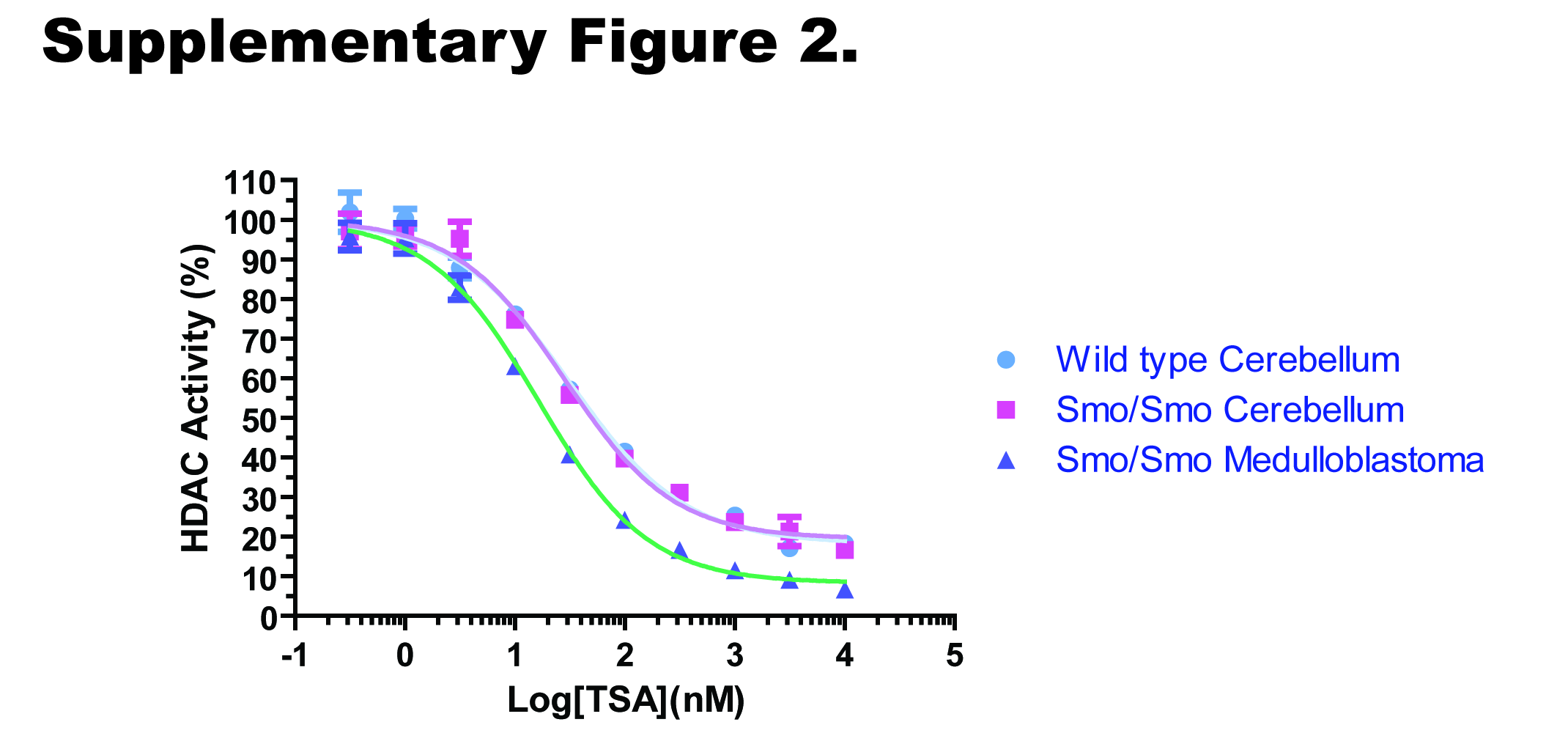

Supplement: Figure S2 — HDAC activity was measured from wild type cerebellum, Smo/Smo cerebellum and Smo/Smo medulloblastoma with increasing concentration of TSA. Error bars represent S.E. (TIF) [file pone.0071455.s002.tif]

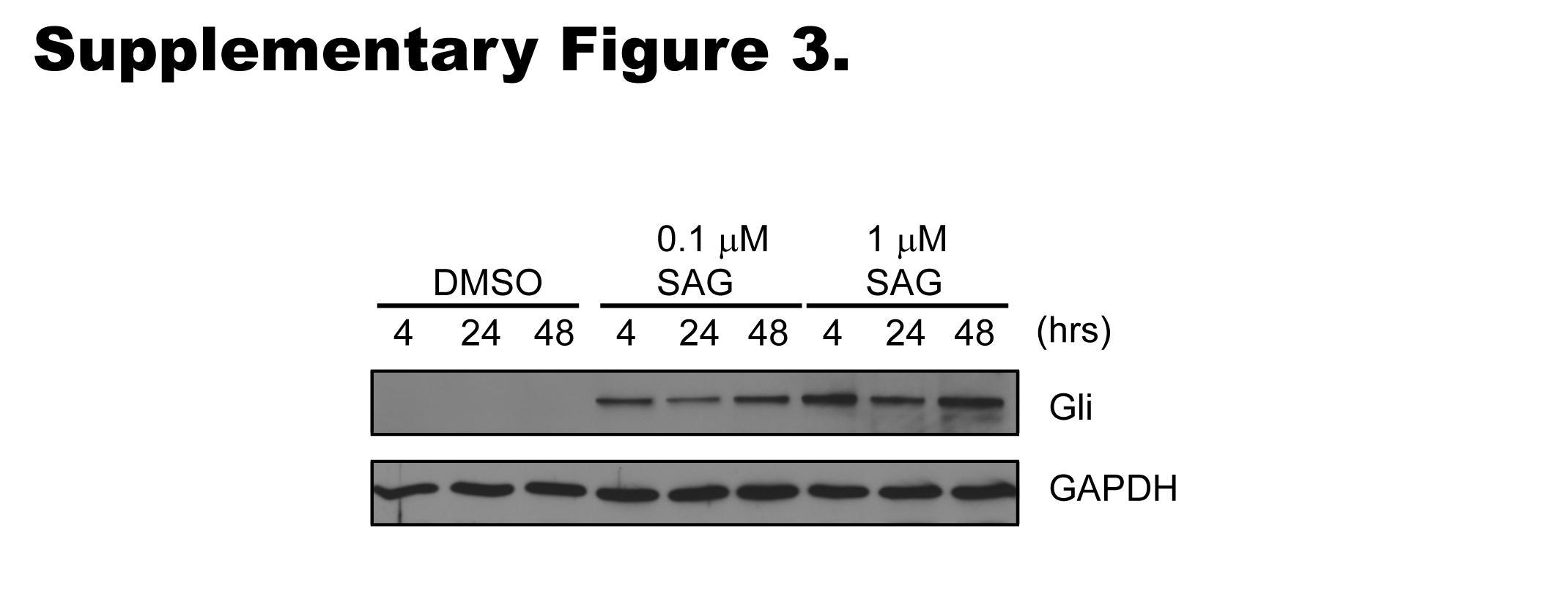

Supplement: Figure S3 — Immunoblot for Gli1 in CGP cells treated with either DMSO or SAG for indicated concentration and time. A GAPDH immunoblot was included as loading control. (TIF) [file pone.0071455.s003.tif]

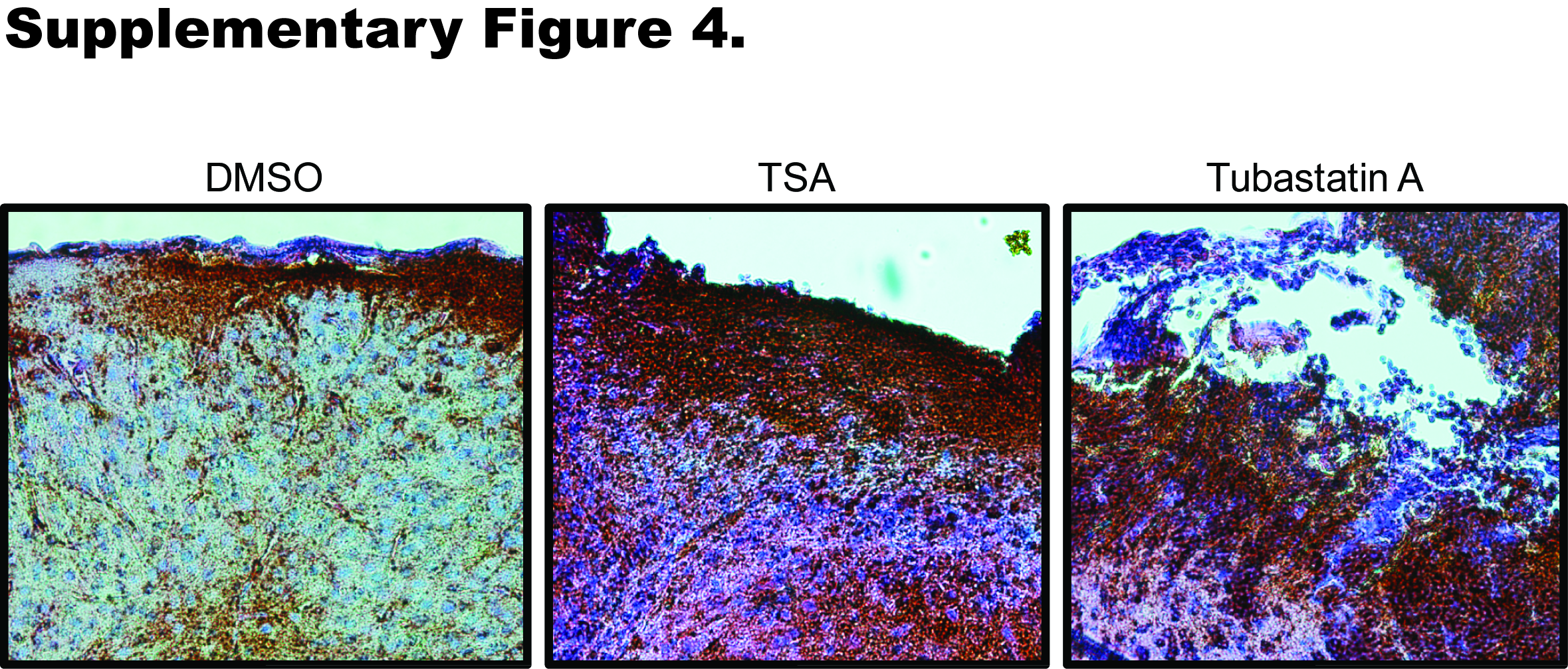

Supplement: Figure S4 — Immunhistochemistry for acetylated tubulin in brain sections from Smo/Smo mice injected with DMSO, TSA or Tubastatin A. (TIF) [file pone.0071455.s004.tif]
