# Supplementary material for: Sonic Hedgehog-Induced Histone Deacetylase Activation Is Required for Cerebellar Granule Precursor Hyperplasia in Medulloblastoma
Source: PLoS One. 2013 Aug 9;8(8):e71455. doi: 10.1371/journal.pone.0071455 (PMC3739791; doi:10.1371/journal.pone.0071455)
Supplement: Table S1 — (DOCX) [file pone.0071455.s005.docx]

**Supplementary table 1. Primer sequences for qPCR.**

| **Species** | **Gene** | **Primer** | **Sequence** | **Amplicon** |
| --- | --- | --- | --- | --- |
| Mouse | HDAC1 | F | TGAAGCCTCACCGAATCCG | 154 bp |
| Mouse | HDAC1 | R | GGGCGAATAGAACGCAGGA |  |
| Mouse | HDAC2 | F | GCTTGCCATCCTCGAATTACT | 114 bp |
| Mouse | HDAC2 | R | GTCATCACGCGATCTGTTGTAT |  |
| Mouse | HDAC3 | F | CCTAGTCCTGCATTATGGTCTCT | 162 bp |
| Mouse | HDAC3 | R | AAGGCATTAAGGCTCTTGGTG |  |
| Mouse | HDAC4 | F | CTGCAAGTGGCCCCTACAG | 177 bp |
| Mouse | HDAC4 | R | CTGCTCATGTTGACGCTGGA |  |
| Mouse | HDAC5 | F | AGCACCGAGGTAAAGCTGAG | 137 bp |
| Mouse | HDAC5 | R | GAACTCTGGTCCAAAGAAGCG |  |
| Mouse | HDAC6 | F | GAGGAGCTGATGTTGGTTCAC | 124 bp |
| Mouse | HDAC6 | R | AGTTCGGATGCAGATACACTGA |  |
| Mouse | HDAC7 | F | AACACTTTCCCTTGCGTAAAACA | 93 bp |
| Mouse | HDAC7 | R | GCAGGGGATTCTTGCGTCT |  |
| Mouse | HDAC8 | F | CACACTGATGCCTATCTGCAA | 101 bp |
| Mouse | HDAC8 | R | GCTGGGCAGTCATAACCTAGTC |  |
| Mouse | HDAC9 | F | CAGAAGCAGCACGAGAATTTGA | 159 bp |
| Mouse | HDAC9 | R | CTCTCTGCGATGCCTCTCTAC |  |
| Mouse | HDAC10 | F | GCTCCACGCACTGTCTAAG | 55 bp |
| Mouse | HDAC10 | R | AAAGGTGTCCGGGTGAAAGTA |  |
| Mouse | HDAC11 | F | CACTGGCTATCAAGTTCCTGTT | 71 bp |
| Mouse | HDAC11 | R | GGCATCGAGATCAATGATGGT |  |
| Mouse | GAPDH | F | CACAGTGGACGACATCCGAAA | 103 bp |
| Mouse | GAPDH | R | AGCTACATAGGAATTACGGGCAA |  |
